# ﻿*Primulajiaozishanensis* (Primulaceae), a new species in *Primula* sect. *Petiolares* subsect. *Davidii* from Yunnan, China

**DOI:** 10.3897/phytokeys.227.103985

**Published:** 2023-05-29

**Authors:** Yuan Wu, Wu-Hai Yang, Zhi-Kun Wu

**Affiliations:** 1 Department of Pharmacy, Guizhou University of Traditional Chinese Medicine, Guiyang, 550025, Guizhou, China Guizhou University of Traditional Chinese Medicine Guiyang China

**Keywords:** Jiao zi shan bao chun, new species, *Primula* sect. *Petiolares*, Yunnan

## Abstract

*Primulajiaozishanensis* Z.K.Wu, W.H.Yang & Yuan Wu, a new species of Primulaceae is described and illustrated from Jiaozi Snow Mountain of Dongchuan District, Yunnan, China. Morphological evidence supports *P.jiaozishanensis* as a member of P.sect.Petiolaressubsect.Davidii, which is characterized by firmly papery or leathery leaves, with veins impressed adaxially, often prominently raised and alveolate abaxially. The new species is characterized by having long and stout rhizomes, smaller leaves with short petioles, short or almost obsolete scape, and larger flowers. The distribution, phenology and conservation status of the new species are also provided.

## ﻿Introduction

*Primula* L. is one of the largest genera in Primulaceae, comprising ca. 500 species worldwide (Hu 1990; POWO 2023). Most of the species occur in temperate and alpine regions of the Northern Hemisphere, e.g., ca. 75% of the total species are found in the Sino–Himalayan region (Hu 1994; Richards 2002). With more than 300 reported species, China has the highest diversity in *Primula*; most of these species are distributed mainly in the southwestern part (Yunnan, Sichuan and Tibet), and the range from the Himalaya–Hengduan mountain chains is the modern diversity center of the genus (Hu 1994; Hu and Kelso 1996; Richards 2002).

The Primulasect.Petiolares Pax is one of the largest sections in Primula ; more than 60 species of this section are now recognized worldwide (Hu 1994; Hu and Kelso 1996; Richards 2002; Hu and Geng 2003; Li and Hu 2009; Rankin 2010; Hu and Hao 2011; Xu et al. 2014, 2016, 2022; Ju et al 2018; Yuan et al. 2018; Wei et al. 2022; Xu et al. 2022; Zhang et al. 2023), and are well represented in the Himalaya–Hengduan mountains, with only a few members extending into Kashmir, central China, and some other regions (Hu 1990; Hu and Kelso 1996). This section was further divided into seven subsections based on the presence or absence of the basal bud scales and farina, the shape of the leaf margin, and the type of hair (Smith and Fletcher 1944). Subsection Davidii is one of the subsections within Primulasect.Petiolares, with recently described species, *P.bergenioides* C.M.Hu & Y.Y.Geng, *P.tenuituba* C.M.Hu & Y.Y.Geng (Hu and Geng 2003), *P.dejuniana* G.Hao, C.M.Hu & Yuan Xu (Xu et al. 2014), *P.wawushanica* G.Hao, C.M.Hu & Yuan Xu (Xu el al. 2016), *P.luteoflora* X.F.Gao & W.B.Ju (Ju et al. 2018), and *P.pingbaensis* Na Zhang, X.Q.Jiang & Z.K.Wu (Zhang et al. 2023). Primulasect.Petiolaressubsect.Davidii comprises 22 species in total, which is characterized by the leaves that are more or less coriaceous, often bullate above and strongly honeycombed–reticulate below, long septate hair more or less clothes the veins, and basal buds covered with paleaceous scales (Smith and Fletcher 1944), mainly distributed in Sichuan, Yunnan and Guizhou in China.

Yunnan is a particularly significant biodiversity hotspot in China, with a wide variety of ecological environments ranging from tropical to alpine subnival belt, possessing ca. 130 species of *Primula* distributed across its range (Fang 2003). With the increased exploration of the region, many new *Primula* species have been discovered and described over the past two decades (Gong and Fang 2003; Xue and Zhang 2004; Shui and Chen 2006; Li and Hu 2009; Hu and Hao 2011; Yang et al. 2017; Wu et al. 2019; Ma et al. 2021; Wang et al. 2022; Wu et al. 2023).

During a botanical expedition to the Jiaozi Snow Mountain in Dongchuan District, Yunnan, southwestern China in May 2017, we found a peculiar population of *Primula* with large flowers, coriaceous leaves persisting into the following spring, basal buds covering paleaceous scales, on a small patch of alpine meadow near the mountain top. For further clarification of the identity of the newly collected *Primula*, the Jiaozi Snow Mountain in Dongchuan District and adjacent areas were revisited in 2020 to observe and collect the plants in flowers. The collected *Primula* is a dwarf perennial herb with a long and stout rhizome, basal buds covered with paleaceous scales, leaves with short petioles and adaxially bullate, short or almost obsolete scape, and large flowers. These features indicate that it should be a member of P.sect.Petiolaressubsect.Davidii. After a full observation of the morphological characteristics and comparing the relevant literature and specimens for related species, we confirmed that this plant represents an undescribed taxon of *Primula*. Therefore, we describe and illustrate the taxon as new to science here.

## ﻿Materials and methods

The morphological observation, measurements and description of the new species were based on living plants from Jiaozi Snow Mountain. Morphological comparison with closely related species was performed based on living plants collected from their type locality, specimens from the key herbaria of China (KUN, PE, WUK), type specimen images online from P, E, K, and relevant literature were also consulted (Smith and Fletcher 1944; Hu 1990; Hu and Kelso 1996). All morphological characters of *P.jiaozishanensis* and its morphologically similar species in the P.sect.Petiolaressubsect.Davidii, including *Primulaesquirolii* Petitm. and *Primulasinoexscapa* C.M.Hu, were measured using a Vernier caliper. The conservation assessment of the new species was evaluated using the IUCN categories of threat (see IUCN 2012 and IUCN Standards and Petitions Committee 2022).

## ﻿Taxonomic treatment

### ﻿ Primula jiaozishanensis

Taxon classificationPlantaeEricalesPrimulaceae

Z.K.Wu, W.H.Yang & Yuan Wu sp. nov.

AB8F5D80-EF81-5FE0-AFCE-5B62831F4DC7

urn:lsid:ipni.org:names:77320210-1

Figs 1 , 2 , 3A, D

#### Diagnosis.

**Figure 1.**
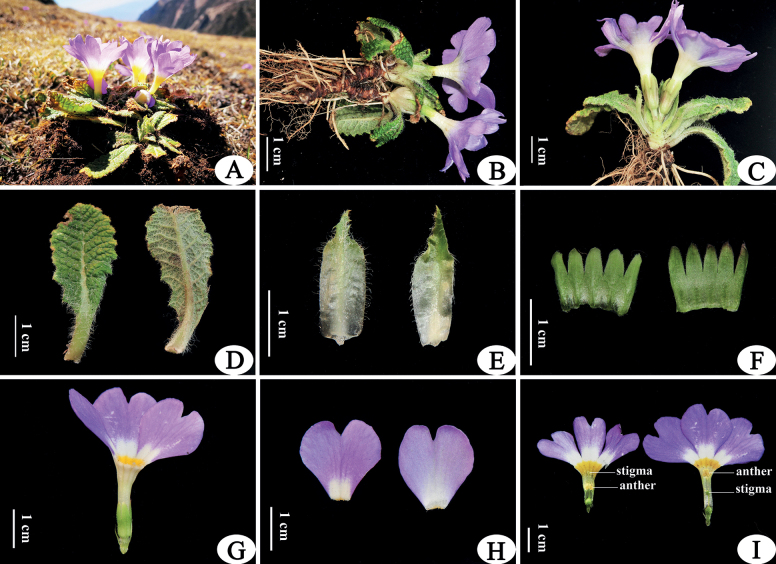
*Primulajiaozishanensis* sp. nov. **A–I A** habitat **B, C** habit in flowering **D** leaves, left: upper surface, right: lower surface **E** bud scales, left: outer surface, right: inner surface **F** calyx, left: outer surface, right: inner surface **G** flower shape **H** corolla lobes, left: upper surface, right: lower surface **I** dissected corolla showing anthers and stigmas, left: pin flower, right: thrum flower. Photographed by Z.K Wu.

**Figure 2.**
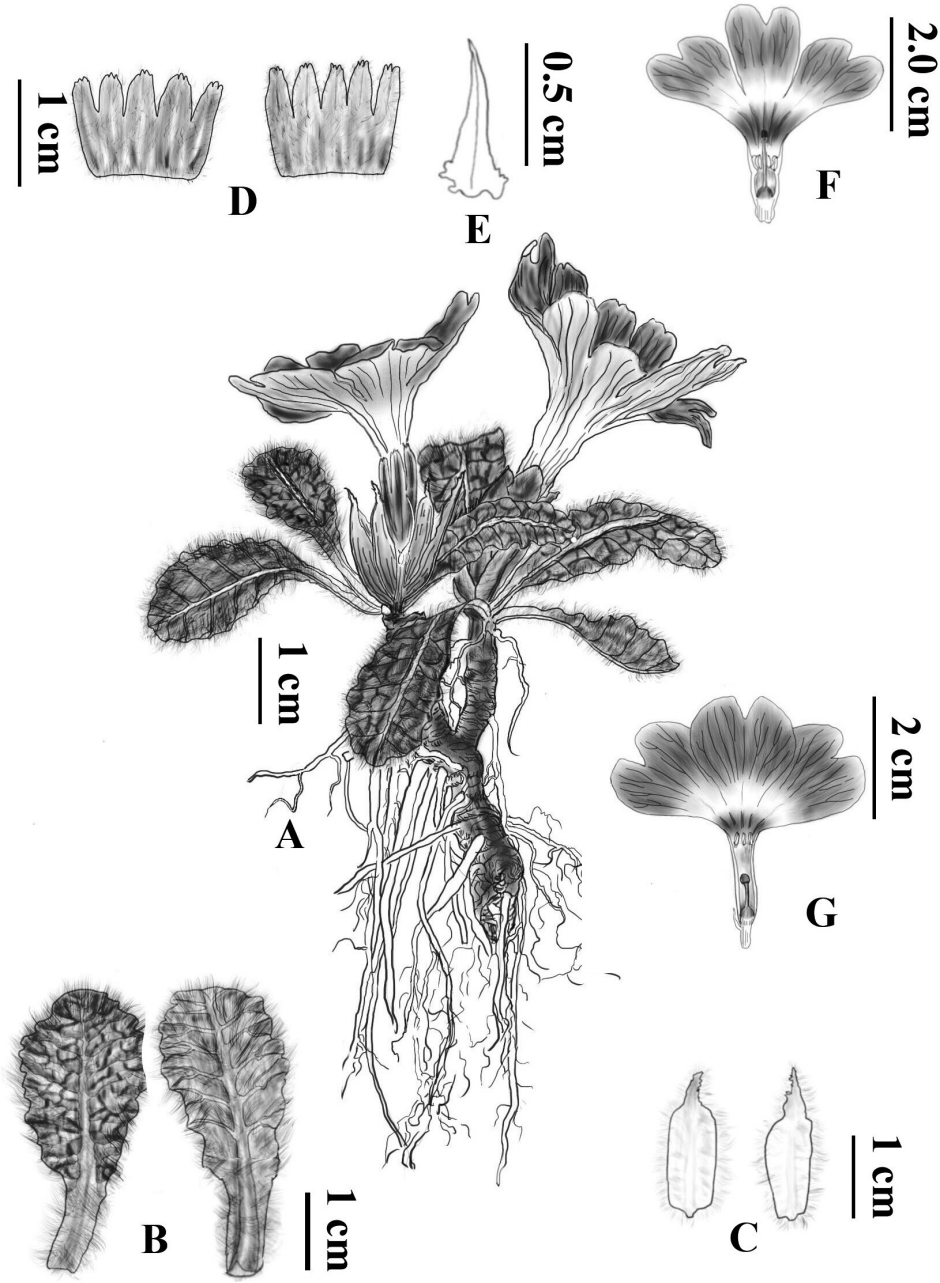
*Primulajiaozishanensis* sp. nov. **A** habit **B** leaves, left: upper surface, right: lower surface **C** bud scales, left: outer surface, right: inner surface **D** calyx, left: outer surface, right: inner surface **E** bract **F** flower, long style (pin) **G** flower, short style (thrum). Drawn by Ms. Xiang–Li Wu.

**Figure 3.**
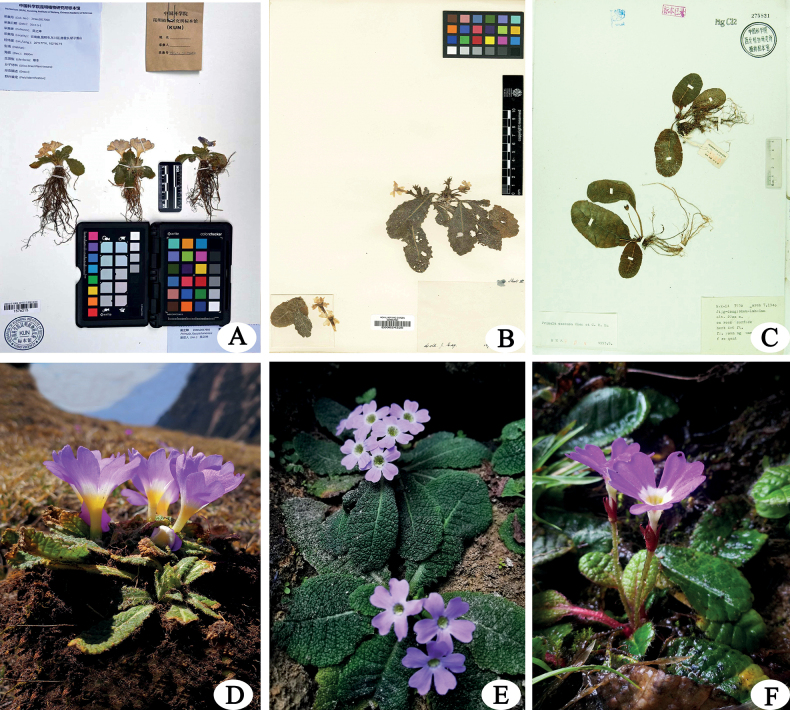
*Primulajiaozishanensis* and two of its allies **A** holotype specimen of *P.jiaozishanensis* (*ZKWU 2017050*, KUN, specimen number KUN1576215) **B** the possible type of *P.esquirolii* (Martin, L. and Esquirol, J., E, specimen number E00024325) **C** paratype of *P.sinoexscapa* from its type locality (*M.K. Li 3530*, WUK, specimen number WUK0275821) **D***P.jiaozishanensis***E***P.esquirolii***F***P.sinoexscapa*. **D–F** photographed by Z.K Wu from their type locality.

The new species most resembles *P.esquirolii* and *P.sinoexscapa*, sharing similar leaves with a more or less rugose surface and short or almost obsolete scape at flowering time. However, the new species differs from the latter two mainly in its long and stout rhizome with 1–3 rosettes, smaller leaf blades with shorter petioles, shorter and stouter pedicels, and larger flowers (Figs 1–3). The main morphological distinctions between *P.jiaozishanensis*, *P.esquirolii* and *P.sinoexscapa* are summarized in Table 1.

**Table 1.** Morphological comparisons of *Primulajiaozishanensis* with *P.esquirolii* and *P.sinoexscapa*.

**Characters**	***P.jiaozishanensis***	***P.esquirolii***	***P.sinoexscapa***
Rhizome	long and stout, 3–4 cm long	short and stout, 1–2 cm long	short and stout, 1–2 cm long
Rosette	1–3	1	1
Scape	almost obsolete or to 1 cm in flowering time	almost obsolete or to 5 cm in flowering time	obsolete in flowering time
Leaf blade	2–4 × 1.5–2.5 cm, spatulate or elliptic–obovate, coriaceous, adaxially covered with sparse white long hairs, abaxially with multicellular hairs along veins.	5–13 × 1.5–5 cm, elliptic–obovate to obovate–oblanceolate, subcoriaceous, abaxially densely short glandular pubescent along veins	2.5–7 (12) × 1.5–3.5 (6) cm, oblong or oblong–obovate, firm papery, abaxially with multicellular hairs along veins, adaxially fulvous pilose along midvein
Petioles	0.5–1.0 cm, thick covering of long multicellular hairs.	short or almost obsolete to 1–2 cm, sparsely glandular.	2–5 cm, with long dense white pilose
Inflorescences	1 flowered or occasionally 2 flowered	2–8 flowered	1–4 flowered
Pedicels	1–3 mm, shorter than bracts	10–20 mm, longer than bracts	15–40 mm, longer than bracts
Calyx	6–8 mm long	5–7 mm long	7–9 mm long
Corolla	tube usually two times as the length of the calyx, limb 30–45 mm wide, lobes obcordate	tube usually 2–3 times as the length of calyx, limb 15–20 mm wide. lobes obovate	tube nearly two time as the length of the calyx, limb 15–20 mm wide, lobes narrowly obovate
Flower	heterostylous	homostylous	heterostylous
Habitat	open alpine meadow	moist limestone cliffs	moist limestone cliffs

#### Type.

China. Yunnan: Dongchuan District, Luoxue xiang, Jiaozi Snow Mountain. 26°9.77'N, 102°56.7'E, 3990 m alt., 1 May 2017 (fl.), *Zhikun WU ZKWu2017050* (holotype: KUN!; isotype: KUN!).

#### Description.

A perennial hairy robust, dwarf herbaceous, efarinose, with a long stout rhizome and numerous fibrous roots, rhizome 3–4 cm long, ca. 0.5 cm in diameter; at flowering time girt at the base by ovate to oblong paleaceous bud scales, scales 1.5–1.8 cm long, 0.4–0.6 cm broad, acute at the apex, outer surface with a sparse covering of long multicellular hairs. ***Leaves*** forming 1–3 rosettes, leaves of current year not well–developed at anthesis, leaves of previous year at flowering time 2–4 cm long including the petiole, 1.5–2.5 cm broad, spatulate or elliptic–obovate, obtuse or rounded at the apex, gradually tapering into the winged petiole; petiole 0.5 to 1 cm, 1/4 as long as leaf blade, stout with a thick covering of long multicellular hairs; lamina coriaceous, adaxially bullate, covered with sparse white long hairs, abaxially with multicellular hairs along veins, margin with regular sparsely acute serrate. ***Scape*** at flowering time almost obsolete or to 1 cm, usually solitary, covered by bud scales, with a thick covering of long multicellular hairs, usually 1 flowered or occasionally 2 flowered. ***Bracts*** linear–lanceolate, 3–6 mm long, glabrous; pedicel 1–3 mm, shorter than bract, with a thick covering of long multicellular hairs. ***Flower*** heterostylous; calyx campanulate, 6–8 mm long, puberulous, parted to 1/2 of its length, lobes ovate to ovate–lanceolate, apex obtuse or occasionally serrate; corolla funnel–shaped, purplish blue or violet, tube 15–20 mm long, usually twice the length of the calyx, limb 30–45 mm wide, lobes obcordate, 13–18 mm long, apex deeply emarginate. ***Pin flowers***: corolla tube 14–16 mm long, stamens ca. 7 mm above the base of the corolla tube, style 14–16 mm long. ***Thrum flowers***: corolla tube 16–20 mm; stamens 16–18 mm above base of corolla tube, style ca. 8 mm long. ***Capsule*** unknown.

#### Distribution and ecology.

**Map 1.**
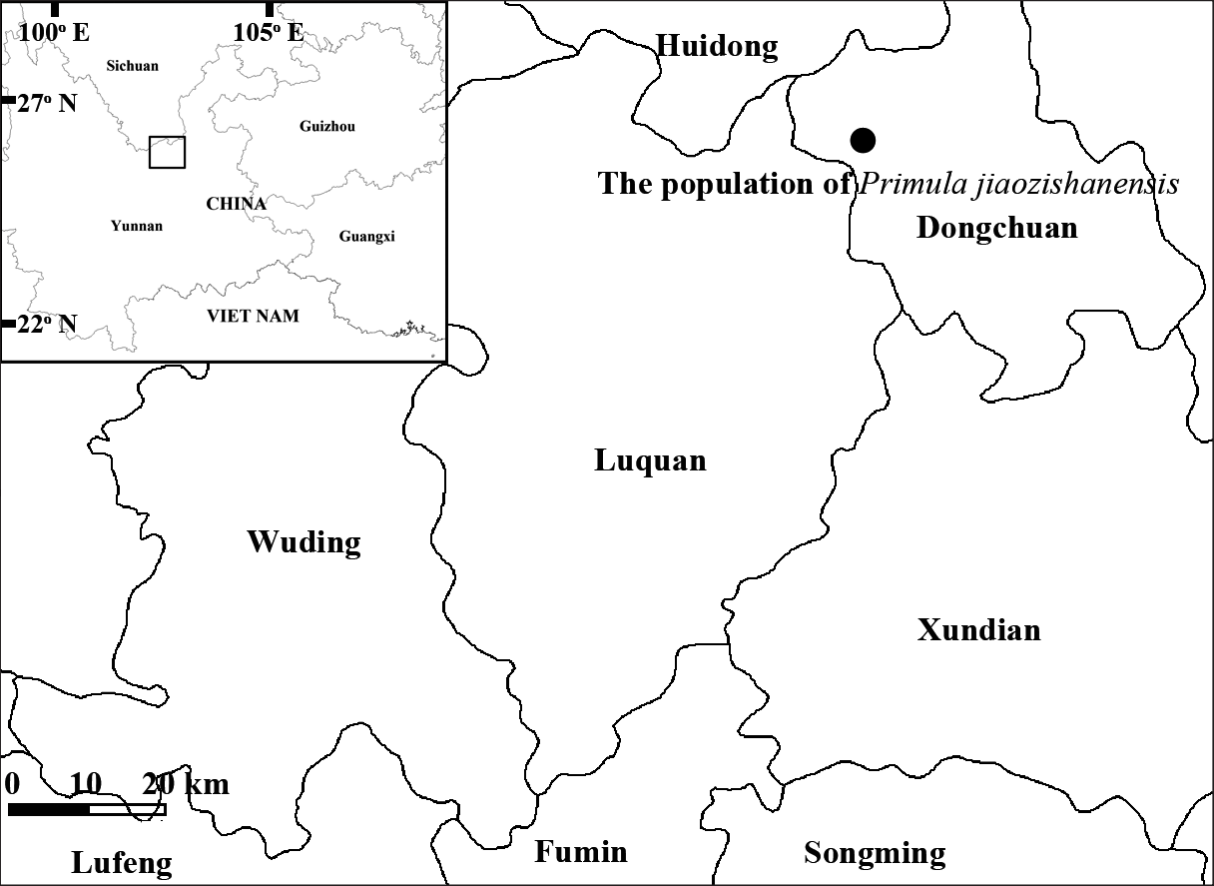
Location of the population of *Primulajiaozishanensis* in Dongchuan District, Yunnan.

*Primulajiaozishanensis* is only known from the type locality on Jiaozi Snow Mountain in Dongchuan District, Yunnan, China. The plant grows in the open alpine meadow (Fig. 1, Map 1).

#### Phenology.

Flowering occurs from April to May.

#### Etymology.

The specific epithet of the new species is taken from the Chinese Pinyin, “Jiaozishan”, the name of the mountain in Northern Yunnan, China, where the type specimen was collected (Map 1).

#### Vernacular name.

Chinese mandarin: *jiao zi shan bao chun* (轿子山报春).

#### Provisional Conservation status.

Critically Endangered (CR B2ab(iii)). The authors have conducted field surveys several times in the type locality and adjacent districts (e.g., Luquan, Qiaojia and Huize) for this new species, and discovered only one population of *Primulajiaozishanensis*, with approximately 100 adult individuals, distributed over about 100 m^2^ in the type locality. This site is in a dry alpine meadow; the new species grows very close to the path for visitors and faces a strong threat from grazing and human activities. Its status should therefore be of concern and addressed by further investigations.

We estimated the extent of occurrence of the species to be less than 10 km^2^. Over the last five years, we have observed a steady decline in the territory area of the habitat due to road construction and grazing. Considering the present field information and IUCN categories of threat (IUCN Standards and Petitions Committee 2022), this species should be included in the category Critically Endangered (CR B2ab(iii)).

#### Additional specimens examined

**(*paratypes*).** The same locality as holotype, 8 May 2020 (fl.), *Zhikun WU ZKWu 2020045* (KUN!).

## ﻿Discussion

The species in Primulasect.Petiolaressubsect.Davidii usually tend to have larger leaves because the plants grow in small groups on shady and moist cliffs beside streams and waterfalls. The leaves of *P.davidii* we found in the field of Sichuan can even reach 30 cm in length, and they also have a short and stout rhizome. The new species *P.jiaozishanensis* grows in open alpine meadow, and its habitat differs from that of other members in the P.sect.Petiolaressubsect.Davidii. Compared to other species in the same subsection, it is distinctive in its long and stout rhizome with 1–3 rosettes, smaller leaf blades, and very short and stout scape with one or occasionally two larger flowers. These features may be an adaptation to the harsh habit of the open alpine meadow, which is usually very windy and has insufficient water in late April and May when it starts anthesis.

## Supplementary Material

XML Treatment for Primula jiaozishanensis

## ﻿Additional information

### Author ORCIDs

Wu-Hai Yang https://orcid.org/0009-0007-6927-8309

Zhi-Kun Wu https://orcid.org/0000-0002-4583-7782

Yuan Wu https://orcid.org/0000-0003-2167-2351

### Data availability

All of the data that support the findings of this study are available in the main text or Supplementary Information.
